# Berberine prevents lethal EV71 neurological infection in newborn mice

**DOI:** 10.3389/fphar.2022.1027566

**Published:** 2022-10-25

**Authors:** Guangyi Cui, Han Wang, Chongting Yang, Xiaoxiao Zhou, Junyi Wang, Tong Wang, Tonghui Ma

**Affiliations:** School of Medicine & Holistic Integrative Medicine, Nanjing University of Chinese Medicine, Nanjing, China

**Keywords:** hand foot and mouth disease, EV71, BBR, neural infection, astrocytes, inflammatory storms, Nrf2, ROS

## Abstract

Enterovirus 71 (EV71) is the major pathogen causing fatal neurological complications of hand, foot, and mouth disease (HFMD) in young children. Currently no effective antiviral therapy is available. In the present study, we found that natural compound Berberine (BBR) displayed potent inhibitory effects on EV71 replication in various neural cells (IC_50_ of 2.79–4.03 μM). In a newborn mouse model of lethal EV71 infection, Berberine at 2–5 mg/kg markedly reduced mortality and clinical scores. Consistently, the replication of EV71 and pathological changes were attenuated in various infected organs including brain and lung with BBR treatment. Interestingly, EV71 infection in the brain mainly localized in the peripheral zone of brainstem and largely in astrocytes. Primary culture of astrocytes from newborn mouse brain confirmed the efficient EV71 replication that was mostly inhibited by BBR treatment at 5 μM. Further investigations revealed remarkably elevated cellular reactive oxygen species (ROS) levels that coincided with EV71 replication in primary cultured astrocytes and various cell lines. BBR largely abolished the virus-elevated ROS production and greatly diminished EV71 replication by up-regulating NFE2 like bZIP transcription factor 2 (Nrf2) *via* the kelch like ECH associated protein 1 (Keap)-Nrf2 axis. The nuclear localization of Nrf2 and expression of downstream antioxidant enzymes heme oxygenase 1 (HO-1) and NAD(P)H quinone dehydrogenase 1 (NQO1) were increased significantly by BBR treatment. Collectively, our findings revealed that BBR prevents lethal EV71 neurological infection *via* inhibiting virus replication through regulating Keap-Nrf2 axis and ROS generation in astrocytes of brainstem, thus providing a potential antiviral treatment for severe EV71 infection associated with neurological complications.

## Introduction

Hand, foot, and mouth disease (HFMD) is an infectious disease caused by various enteroviruses, which is a severe public health problem threatening children under 5 years (Cox and Levent, 2018). Enterovirus 71 (EV71), the most common pathogen causing HFMD, belongs to the Picornaviridae family, genus Enterovirus, Enterovirus species A. It is a non-enveloped RNA virus containing a single-stranded, sense-strand, polyadenylated RNA of approximately 7,400 nucleotides (Solomon et al., 2010). HFMD is normally self-limiting, typical HFMD manifest with mild flu-like symptoms and usually disappear 7–10 days after disease onset. However, a small proportion (1.1%) of the patients rapidly develop neurological and systemic complications that can be fatal, such as aseptic meningitis (AM), brain stem encephalitis (BSE), acute flaccid paralysis (AFP), neurogenic pulmonary edema (NPE), cardiopulmonary failure, and even lethal encephalitis in neonates (Ooi et al., 2010; Huang and Shih, 2014; Xing et al., 2014; Gonzalez et al., 2019; Tan and Chu, 2021). Pulmonary edema and pulmonary hemorrhage due to neurological damage are considered major causes of death from EV71 infection. More than seventy percent of severe HFMD and ninety-three percent of fatal HFMD cases confirmed in the laboratory were associated with EV71 infection (Xing et al., 2014). Since the late 1990s, large EV71 outbreaks have repeatedly occurred in Asian countries and hundreds of cases involving lethal complications have been reported in each outbreak (Chen et al., 2007; Xing et al., 2014; Liu et al., 2015; Gonzalez et al., 2019; Van et al., 2019). For example, more than 7 million HFMD cases were reported in China between 2008 and 2012, of which 2,457 were fatal (Xing et al., 2014). Millions of children are infected with EV71 each year over the world, and the morbidity and mortality of HFMD have increased annually, such as recent (2018) epidemic in Vietnam, Singapore and Malaysia where more than 100,000 children were hospitalized, emphasizing the severity of disease progression and the urgency to develop antivirals (Nhan et al., 2018). However, the mechanisms underlying neurological pathogeneses of EV71 infection remain largely unclear, leading to the lack of approved antiviral agents against EV71 fatal infection. Ribavirin is a broad-spectrum antiviral drug that was reported to inhibit EV71 infection, but it is not licensed by the FDA because its clinical effect is unclear (Li et al., 2008). Therefore, it is important to search for effective antiviral therapy for EV71 infections, especially fatal infection caused by severe neurological complications.

By screening of a natural product library from Selleck Chemicals, we identified Berberine (BBR) as a potent inhibitor of EV71 cytopathic infection in various human cell lines, preferentially in neural type cells. BBR is a naturally derived isoquinoline alkaloid with multiple pharmacological effects and widely used in Asian countries (Wang K. et al., 2017; Feng et al., 2019). It has been reported to have broad antiviral activities against human immunodeficiency virus, dengue virus, zika virus, human cytomegalovirus, human papilomavirus, hepatitis C virus, chikungunya virus, human influenza virus, respiratory syncytial virus, herpes simplex virus, SARS-CoV-2, and enteroviruses (Hayashi et al., 2007; Mahata et al., 2011; Song et al., 2014; Shin et al., 2015; Warowicka et al., 2020; Varghese et al., 2021). In addition, BBR has been shown to cross the blood-brain barrier efficiently and exhibited neuroprotective effects in many neurological disorders (Kulkarni and Dhir, 2010; Cheng et al., 2022). Therefore, BBR may hold promising efficacy for neuronal injury caused by EV71 infection. In the present study, we explored the *in vivo* efficacy and underlying mechanisms of BBR in protecting the fatal EV71 neurological infection in a newborn mouse model.

## Materials and methods

### Cells and virus

U251 (09063001, European Collection of Cell Cultures, United Kingdom), SK-N-MC (HTB-10, American Type Culture Collection (ATCC), United States) and A549 (CCL-185, ATCC, United States) cell lines were cultured in DMEM or MEM medium (Gibco) containing penicillin-streptomycin (1% v/v) and fetal bovine serum (FBS, 10%; Gibco) at 37°C in an atmosphere of 5% CO_2_.

Wild-type EV71 C4 strain (Gene Bank accession no. KJ508817) was obtained from Chinese Center for Disease Control and Prevention (Beijing, China). EV71 C4 stocks were produced in A549 cells and preserved at −80°C. Virus titer (TCID_50_) was calculated by the Reed-Muench method as described previously (Fu et al., 2017; Yu et al., 2021).

### Chemicals and reagents

BBR (SB8130), Ribavirin (SR8570), N-Acetylcysteine (NAC, IA0050). All compounds were more than 98% pure by HPLC.

### Animals

Pregnant mice (ICR) were purchased from Qinglongshan Animal Breeding Center (Nanjing, China). All animal experiments were approved by the Experimental Animal Ethics Committee of Nanjing University of Chinese Medicine (202201A034). The newborn mice (born within 24 h from the breeder mice) were selected as subjects of *in vivo* experiments.

### Antiviral screening of natural product

A natural product library (L1400, Selleck, United States) was screened at the dose of 50 µM in EV71-infected U251 glioma cells (MOI = 0.1) in 96-well plates. Compounds were added to the cells immediately after EV71 infection, and cell viability was measured by Cell Counting Assay kit (CCK-8, Meilunbio, China) at 72 h post infection to determine the antiviral activity as described previously (Yu et al., 2021).

### Cytotoxicity and cytopathic effect inhibition assays

Cells were seeded in 96-well plates overnight. BBR (400–25 μM) or ribavirin (400–25 μM) was added to cells at different concentrations. CCK-8 was used to measure the cell viability after 72 h incubation according to the manufacturer’s directions. The 50% cytotoxic concentration (CC_50_) of compounds was determined by linear regression analysis.

To determine the cytopathic effect (CPE) inhibition activity, cells were treated with different concentrations of BBR (25–1.5625 μM) or ribavirin (200–12.5 μM) after infection (MOI = 0.1). Cell viability was measured by CCK-8 at 72–96 h post infection to calculate the CPE inhibition rate as reported (Yu et al., 2021). The 50% effective concentration (IC_50_) of compounds was calculated by linear regression of the CPE inhibition curves.

### Time of compound addition assay

The assay was carried out as reported previously with some modifications (Dai et al., 2017). Cells were seeded in 12-well plates. After infection with EV71 (MOI = 1), the cells were treated with 5 μM BBR before (PRE: 5-0 h), simultaneous (SIM: 0–1 h), post infection (POST: 1–5 h) and late post infection (Late POST: 5–16 h) to determine the stage of inhibitory effects in virus life cycle. To detect whether BBR inactivated EV71 directly, EV71 and BBR were mixed and incubated at 4°C for 5 h before infection (DIR). Moreover, to further investigate the efficacy of consecutive administrations, BBR was also maintained until 16 h post infection in addition to the above procedures. The EV71 RNA was extracted from the cells and quantified by qPCR after the cells were incubated for 16 h after infection.

### Western blotting

Protein samples were prepared using RIPA buffer (Beyotime, China), resolved by SDS polyacrylamide gel electrophoresis and transferred to PVDF membranes. The blots were incubated with primary antibodies for (Keap1, 1:2000, 10503-2-AP, Rabbit; HO-1, 1:2000, 10701-1-AP, Rabbit; NQO1, 1:2000, 11451-1-AP, Rabbit; GAPDH, 1:5000, 10494-1-AP, Rabbit; β-Tubulin, 1:4000, 10094-1-AP, Rabbit; β-actin, 1:2000, 20536-1-AP, Rabbit) (Proteintech, China) and for Nrf2 (1:1000, ab137550, Rabbit; Abcam, United Kingdom) and EV71 VP1 (1:2000, GTX132338, Rabbit; GeneTex, United States) at 4°C for 16 h, followed by the addition of horseradish peroxidase-conjugated goat anti-Rabbit (1:5000, 7,074) IgG secondary antibodies (CST, United States) for 2 h at room temperature. Positive bands were detected by chemiluminescence.

### Quantitative real-time PCR and siRNA transfection

RNA was isolated from cells or tissues using TRIzol reagent (Invitrogen™, United States). The transcripts of various genes were quantified using SYBR Green master mix (YeaSEN, China) following the manufacture’s protocol. Gene transcript levels were determined by the ΔΔCT method. Nrf2 siRNA (TGC​TCA​GAA​TTG​CAG​AAA​A) and Keap1 siRNA (GGA​GGU​GGU​GUC​CAU​UGA​ATT) were purchased from GenePharma (Shanghai, China) and transfected into cells following the manufacturer’s instructions.

### Immunofluorescence

The cells or tissues were fixed with 4% paraformaldehyde for 30 min, permeabilized using 0.25% Triton X-100 for 15 min, blocked with 1% BSA for 1 h and incubated with primary antibodies (EV71, 1:200, ab36367, Mouse; Glial fibrillary acidic protein (GFAP), 1:100, ab7260, Rabbit; Allograft inflammatory factor 1 (IBA-1), 1:100, ab153696, Rabbit; RNA binding fox-1 homolog 3 (NeuN), 1:100, ab177487, Rabbit; Microtubule associated protein 2 (MAP2), 1:100, Rabbit; Nrf2, 1:200, ab137550, Rabbit) overnight at 4°C. After washing the slides were incubated with the fluorescence-conjugated goat anti-Rabbit (1:500, SA00013-2) or anti-Mouse (1:500, SA00013-3) IgG (H + L) antibodies (Proteintech, China) as secondary antibodies for 1 h at 37°C. Then nuclear staining was done with DAPI. Images were captured using laser-scanning confocal or thunder fluorescence microscope (Leica, Germany). Fluorescence intensity of nucleus and cytoplasm was analyzed using Columbus (an image data storage and analysis server) and High Content Image Processing and Analysis System (Operetta CLS, PerkinElmer, United States).

### Measurements of ROS

Cellular ROS production was examined using the 2ʹ,7ʹdichlorofluorescein diacetate (DCFH-DA) (Beyotime, China). 24 h after infection with EV71, the cells were incubated with DCFH-DA (10 µM) for 30 min in the dark at 37°C. Fluorescence intensity was assessed using fluorescence microscope (Leica) and C6 Plus flow cytometer (BD).

### Enzyme-linked immunosorbent assay

The protein levels of IL-6 (AB-5737, Abmart, China), TNF-α (AB-J0225, Abmart, China) and IL-1β (MLB00C, Novus, China) were determined using ELISA kits following the manufacturer’s instructions.

### *In vivo* anti-EV71 efficacy in newborn mice

Newborn ICR mice were infected with 10^6^ TCID_50_ (lethal dose) of EV71 by intraperitoneal injection as reported previously with some modifications (Lee et al., 2014; Dai et al., 2017). After 2 h, the pups were intraperitoneally injected BBR (2–5 mg/kg) or ribavirin (20 mg/kg) in PBS supplemented with 10% DMSO and 40% Propylene Glycol for 7 consecutive days. Control group were injected with equal volume of the solvent. The mice were monitored daily for clinical scores, body weight and mortality.

### Primary culture of astrocytes

Primary culture of mouse astrocytes was performed as described previously with some modifications (O'Sullivan et al., 2017; Velasco-Estevez et al., 2020). Briefly, the brains of ICR newborn mice (within 24 h) were separated under an anatomical microscope, shredded with ophthalmic scissors, digested with 0.5% trypsin for 15 min, then centrifuged and resuspended in DMEM (Gibco) supplemented with 10% FBS (Gibco) and 1% penicillin-streptomycin for culture. After 24 h, the medium was replaced and 1 μM cytarabine was added to inhibit the growth of other cells. After 14 days, flasks containing cell cultures were shaken for 2 h at 200 rpm using an orbital shaker to eliminate most of the microglia and oligodendrocytes. The enriched astrocyte culture was exposed to 0.5% trypsin for 5 min to detach the glial monolayer and cells were re-plated in a new flask to further purify the astrocytes culture by eliminating microglia. The astrocytes culture is >95% pure as determined by GFAP immunostaining.

### Statistical analysis

The data are expressed as the mean ± standard deviation (SD). Statistical differences were analyzed by Student’s t-test or analysis of variance (ANOVA). A value of *p* < 0.05 was considered statistically significant. Statistical significance represented by asterisks was marked correspondingly in the figures, where *p < 0.05, **p < 0.01, ***p < 0.001.

## Results

### Berberine suppresses cytopathic effect of EV71 by inhibiting virus replication

**FIGURE 1:**
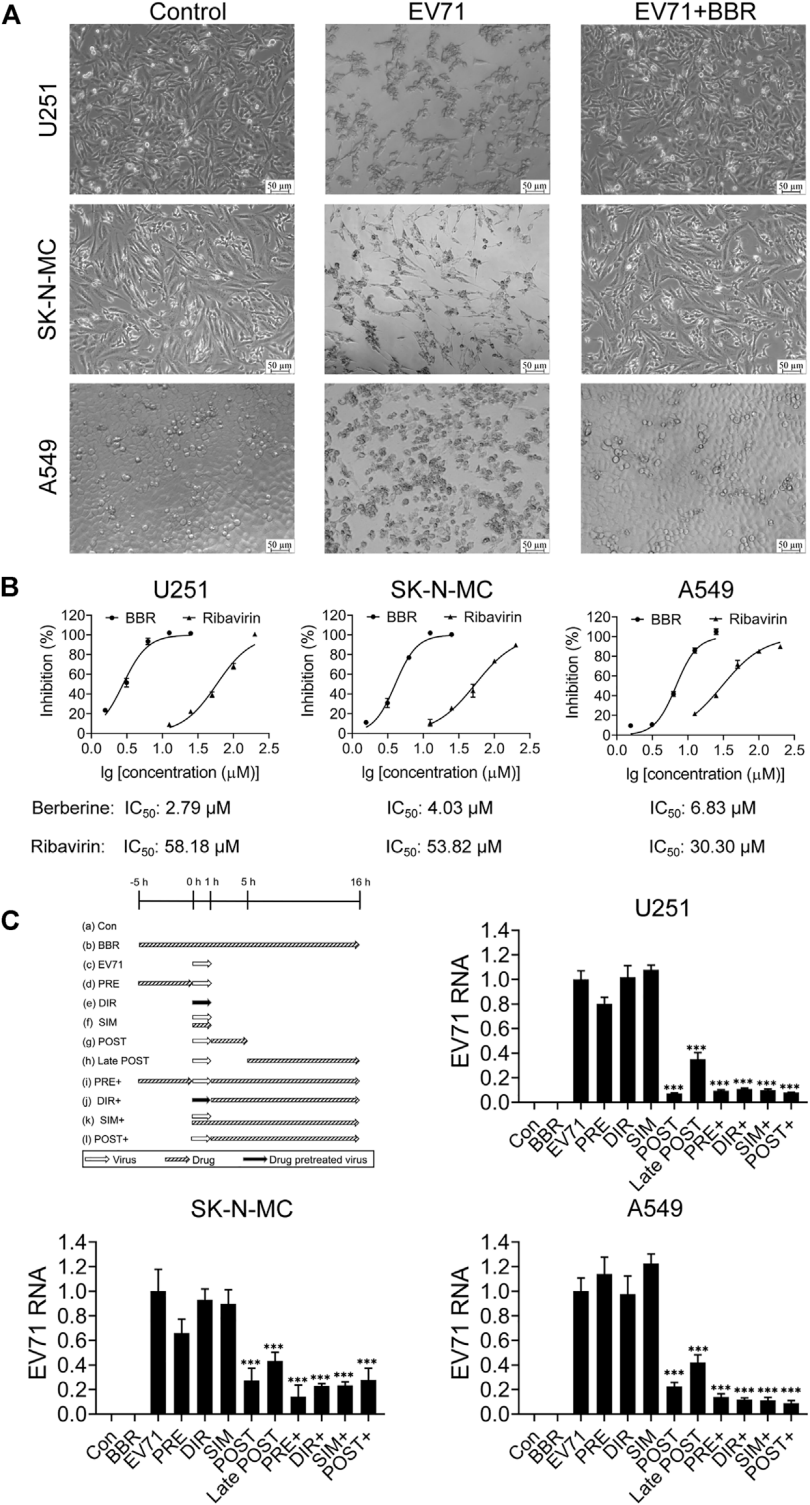
Antiviral activities of BBR against EV71. **(A)** Morphological changes of U251, SK-N-MC, and A549 cells at 72 h post EV71 infection w/o BBR treatment in the CPE inhibition assay. Bar = 50 μm. **(B)** Dose-dependent anti-EV71 activities of BBR and ribavirin in different cell lines. **(C)** Effects of BBR on EV71 replication in the time of compound addition assay in different cell types. BBR concentrations at 5 μM (U251) or 10 μM (SK-N-MC and A549) were used (*n* = 6). Data are presented as SD. Statistical significance was compared between the test groups and the EV71 group was represented by asterisks marked correspondingly in the figures, ***p < 0.001 vs. EV71 group.

Three human cell lines U251, SK-N-MC and A549 were infected with EV71 in the presence or absence of BBR to explore the antiviral potential of BBR against EV71. As shown in Figure 1A, BBR at 5 μM (U251) and 10 μM (SK-N-MC and A549) concentration largely reversed the CPE caused by EV71 infection. Figure 1B shows the dose-dependent antiviral activities of BBR against EV71 with ribavirin (a broad-spectrum antiviral drug reported to be effective in patients with hand-foot and mouth disease in recent research (Dai et al., 2017)) as positive control. The IC_50_ value of BBR on U251, SK-N-MC and A549 cells were 2.79 μM, 4.03 μM and 6.83 μM respectively, much more potent than ribavirin with IC_50_ of 58.18 μM, 53.82 μM and 30.30 μM in the 3 cell lines. In addition, CCK-8 assay in U251, SK-N-MC and A549 cells showed no obvious cytotoxicity at concentrations lower than 100 μM (Supplementary Figure S1).

The single lifecycle of EV71 is about 16 h, in which 0–1 h is the adsorption and entry stage, 1–5 h is the replication stage, and 5–16 h is the assembly and release stage (Dai et al., 2017). To identify the target stage of EV71 life cycle by BBR, we analyzed the effect of BBR added at different time frames before or after virus infection. As shown in Figure 1C, when BBR exists during 1–5 h after virus infection, the cellular EV71 RNA contents decreased by >60% compared with EV71 group. And the late-post BBR treatment (5–16 h) reduced EV71 RNA level by ∼50%. The results clearly indicated that BBR prevents EV71 infection by targeting the replication stage.

### Berberine protects newborn mice from lethal EV71 challenge

**FIGURE 2:**
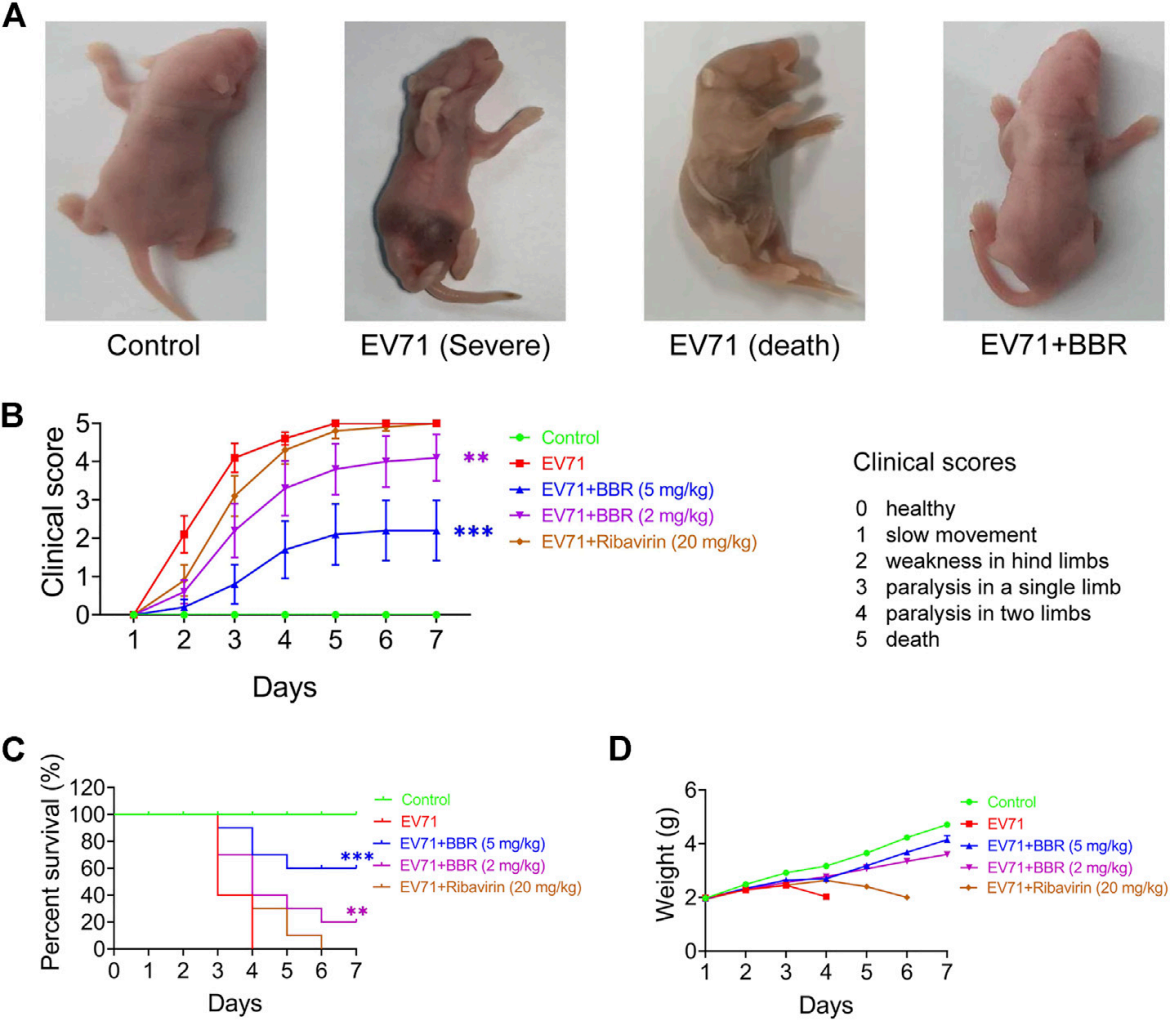
*In vivo* efficacy of BBR against lethal EV71 infection in newborn mice. **(A)** The general condition of normal, EV71-infected (Severe and Death) and BBR treated newborn mice. **(B–D)** Clincal scores, survival rates and body weight changes of the infected and BBR-treated newborn mice (*n* = 10). Data are presented as SD. Statistical significance was compared between the test groups and the EV71 group was represented by asterisks marked correspondingly in the figures, **p < 0.01, ***p < 0.001 vs. EV71 group.

Next we studied the protective efficacy of BBR against EV71 lethal infection in one-day-old ICR mice with ribavirin as positive control (Wei et al., 2014). BBR dosage of 2 mg/kg and 5 mg/kg body weight was selected based on the *in vitro* antiviral activity, previously reported animal studies and clinical trials of the drug (Zhou and Zhou, 2010; Lan et al., 2015; Dong et al., 2016). Figure 2A shows the general looking of control, infected (in severe condition or dead) and BBR-treated newborn mice infected with lethal dose of EV71 (10^6^ TCID_50_). Compared with normal newborn mice, lethal infection caused obvious symptoms such as cyanosis, congestion, paralysis of the limbs and death (Figure 2B), which are consistent with previously reported symptoms in EV71 lethal infection mouse models and clinically severe patients (Zeng et al., 2012). BBR improved the clinical scores significantly at 2 mg/kg and remarkably at 5 mg/kg dosages compared with the minimal protective effects of ribavirin at 20 mg/kg. In addition, BBR dose-dependently increased the survival rate and prevented body weight loss of infected mice with better efficacy than ribavirin (Figures 2C,D). These results establish a protective effect of BBR on lethal EV71 infection in newborn mice.

### Berberine reduced virus replication and pathological injury in EV71 infected organs of newborn mice

**FIGURE 3:**
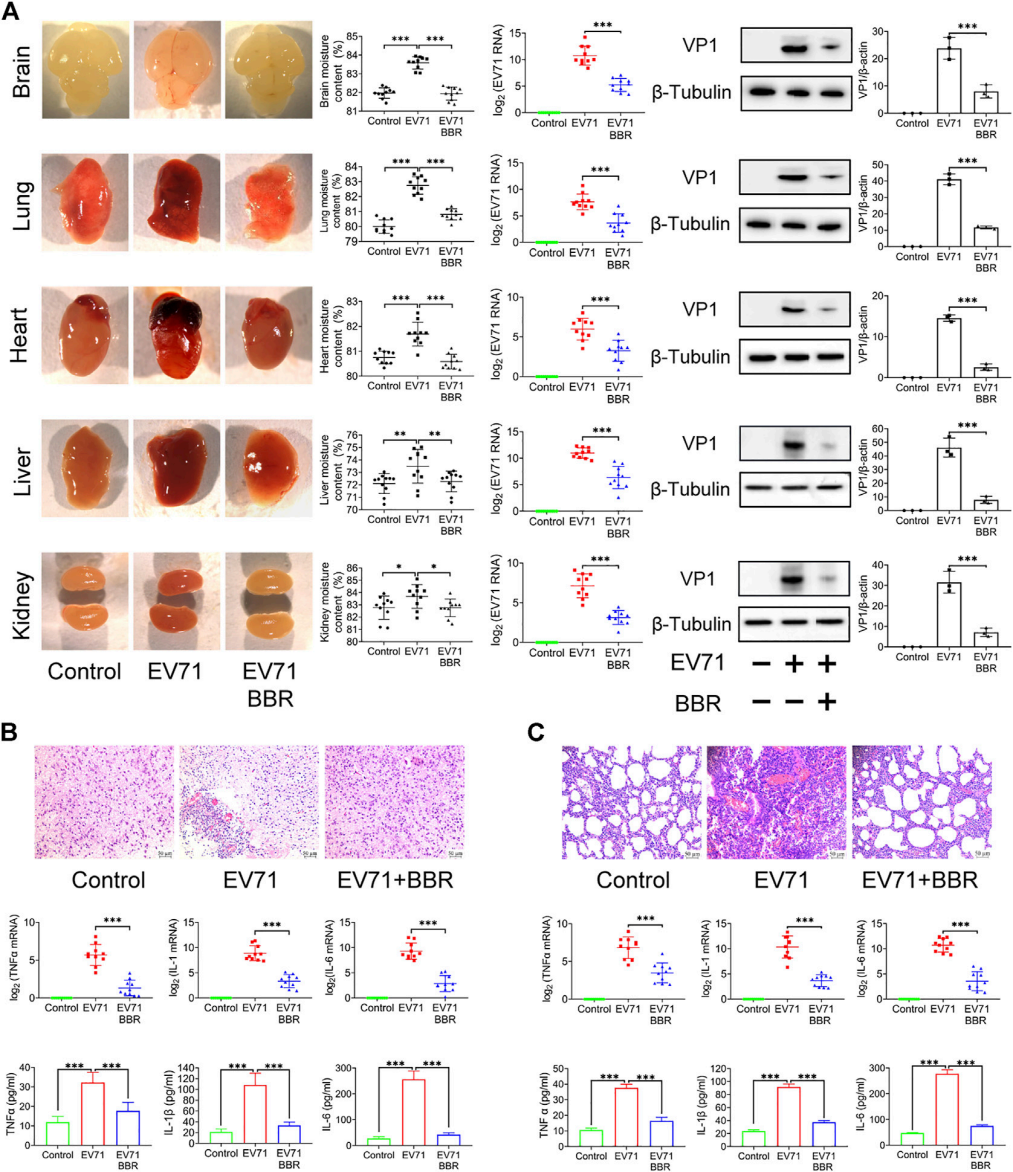
Reduced virus replication and pathological injuries in EV71-infected organs after BBR treatment. **(A)** Effects of BBR (5 mg/kg) on EV71 replication and organ pathology in newborn mice. The moisture content of tissues was detected (*n* = 10). EV71 RNA (copies) and VP1 protein expression levels were determined by qPCR (*n* = 10) and Western blot analysis (*n* = 3). **(B)** and **(C)** Effects of BBR on histopathological injury and expression of proinflammatory factors of brain and lung of the newborn mice. TNFα, IL-1β,and IL-6 mRNA and protein levels were determined by qPCR (*n* = 10) and ELISA kits (*n* = 6). Data are presented as SD. Statistical significance was compared between the test groups and the EV71 group was represented by asterisks marked correspondingly in the figures, ***p < 0.001 vs. EV71 group.

As shown in Figure 3A, we observed congestion, redness and swelling in brain, lung, heart, liver and kidney after EV71 lethal infection, which coincided with overt virus replication indicated by EV71 RNA content and VP1 protein expression in these organs. BBR at 5 mg/kg greatly reduced virus replication and improved the pathological changes. Since neurogenic pulmonary edema caused by brain inflammation and injury is the major cause of death in patients with fatal EV71 infection, we analyzed the effects of BBR on the histopathological changes and pro-inflammatory factors in the brain and lung of newborn mice 3 days after lethal EV71 infection. As shown in Figure 3B, EV71 infected brain exhibited obvious vascular congestion, hemorrhage and inflammatory cell infiltration in the peripheral zone of brainstem, together with remarkably increased RNA and protein expression of pro-inflammatory factors TNFα, IL-1β and IL-6 in infected brainstem. BBR significantly reduced the expression of the pro-inflammatory factors and alleviated the pathological changes. Likewise, BBR markedly decreased the RNA and protein expression of TNFα, IL-1β and IL-6, and ameliorated pathological injury in infected lung (Figure 3C). These results demonstrated that BBR reduced virus replication and inflammatory injury in the key organs of newborn mice in fatal EV71 infection.

### Berberine inhibits EV71 replication in astrocytes in mouse brain

**FIGURE 4:**
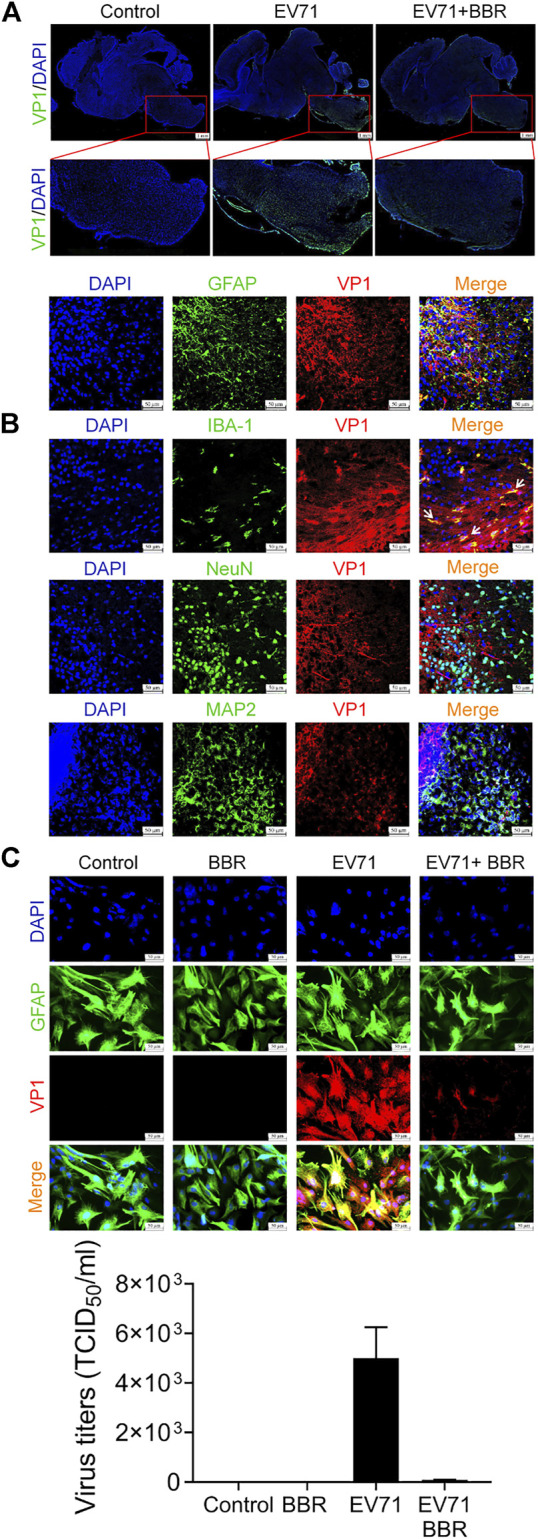
BBR inhibits EV71 replication in astrocytes in mouse brain. **(A)** Effects of BBR (5 mg/kg) on EV71 infection in the brainstem of newborn mice (48–72 h after infection). Longitudinal cryosections of brain containing brainstem were subjected to immunofluorescence staining with DAPI (Blue) and EV71 VP1 protein (Green). Bar = 1 mm. **(B)** Colocalization of EV71 VP1 protein (Red) with GFAP, IBA-1, NeuN or MAP2 (Green) in brainstem by immunofluorescence. Bar = 50 μm. **(C)** Effects of BBR (5 μM) on EV71 infection (MOI = 1) in primary cultured astrocytes. The cells were subjected to immunofluorescence staining with GFAP (Green), VP1 protein (Red) and DAPI (Blue) 24 h after virus infection, Bar = 50 μm. EV71 titers was detected 24 h after virus infection. Data are presented as SD. Statistical significance was compared between the test groups and the EV71 group was represented by asterisks marked correspondingly in the figures, ***p < 0.001 vs. EV71 group.

We further examined the distribution of EV71 infection in mouse brain in the lethal infection model. As shown in Figure 4A, immunofluorescence analysis of EV71 VP1 protein indicated intensive staining in the brainstem and much weaker staining in other parts of the brain. VP1 staining is relatively stronger in the ventral zone of brainstem, where the pathological changes are more obvious (Figure 3B). BBR remarkably inhibited EV71 infection in brainstem. To determine the cell type selectivity of EV71 infection in brainstem, we performed immunofluorescence co-localization of EV71 VP1 protein with different neural cell markers. The viral VP1 protein co-localized with nearly all astrocytes marker GFAP and microglia marker IBA-1, and only occasionally with neuronal marker NeuN and MAP2, suggesting that EV71 preferentially replicates in glial cells in the newborn mouse brain (Figure 4B). It is notable that GFAP-positive cells are more distributed in the peripheral zone of brainstem, which is consistent with the more severe virus infection and pathological changes described above. By primary culture of astrocytes from newborn mouse brain, we confirmed the efficient replication of EV71 by VP1 immunofluorescence and EV71 titers, which was mostly inhibited by BBR at 5 μM (Figure 4C). These data indicated that EV71 neural infection is mainly localized in glial cells in brainstem where BBR efficiently inhibited EV71 replication.

### Berberine inhibits ROS production in EV71 infected cells

**FIGURE 5:**
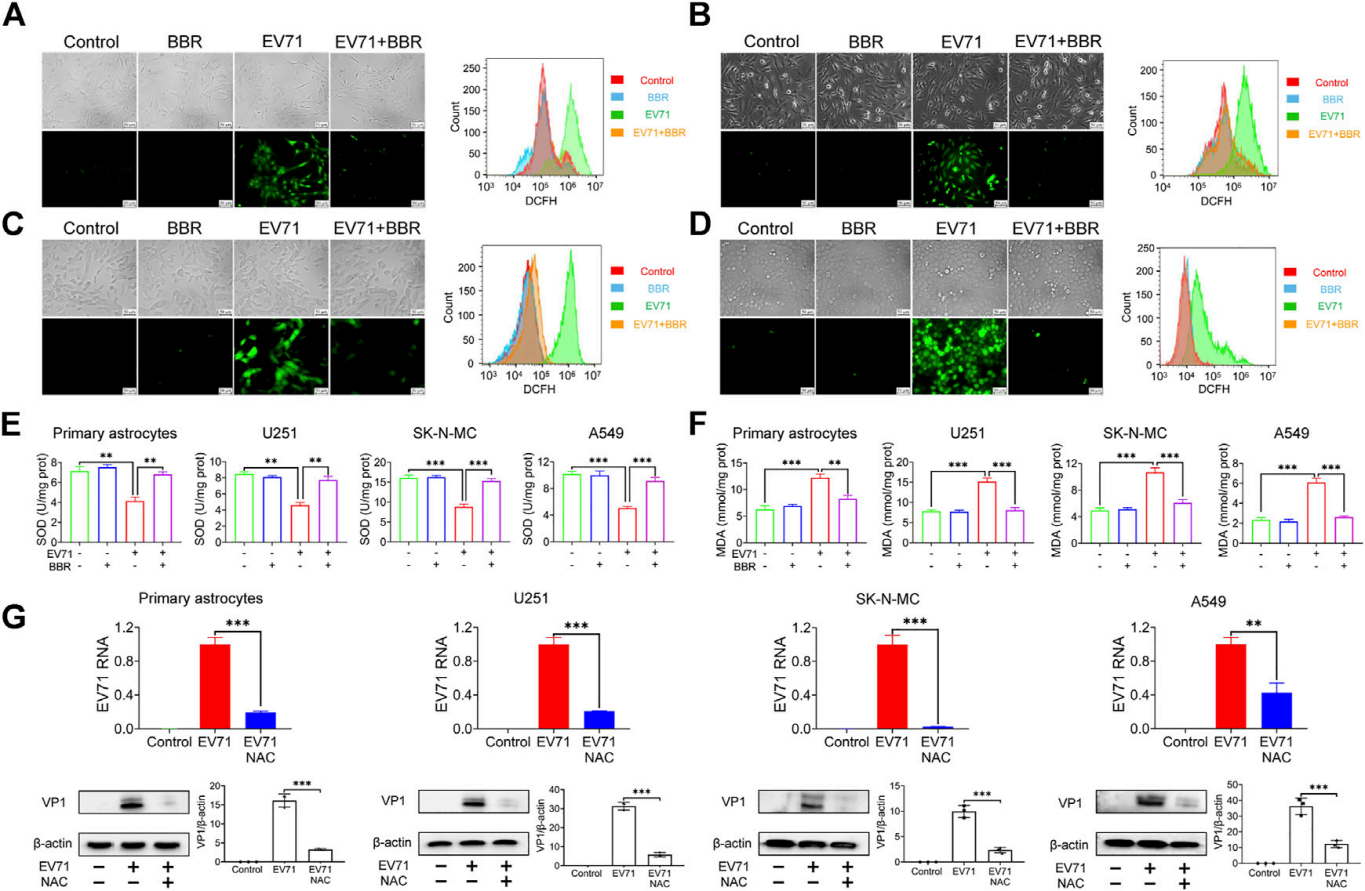
Effects of BBR on EV71-induced cellular ROS production. **(A–D)** ROS production in primary astrocytes, U251, SK-N-MC, and A549 cells with indicated maneuvers was detected using DCFH-DA by fluorescence microscopy (*left*) and FITC flow cytometry (*right*). Cells were infected with EV71 (MOI = 1) for 2 h, then treated with BBR (5 μM for primary astrocytes and U251; 10 μM for SK-N-MC and A549) for 24 h **(E)** and **(F)** Effects of BBR on SOD and MDA levels in cells infected for 24 h. **(G)** Effects of NAC on EV71 replication in the indicated cells. The cells were infected with EV71 (MOI = 1) for 2 h, and then treated with NAC (100 μM) for 24 h. EV71 RNA was quantified by qPCR (*n* = 6) and VP1 protein was detected by Western blot. Data are presented as SD. Statistical significance was compared between the test groups and the EV71 group was represented by asterisks marked correspondingly in the figures, **p < 0.01, ***p < 0.001 vs. EV71 group.

Previous studies indicated that EV71-induced oxidative stress plays a crucial role in its replication (Li H. et al., 2020; Bai et al., 2020). In view of the well-known antioxidant property of BBR (Li W. et al., 2020), we analyzed the intracellular ROS level 24 h after EV71 infection in the presence or absence of BBR. Indeed EV71 infection induced a robust production of ROS in primary astrocytes (Figure 5A) and 3 cell lines U251 (Figure 5B), SK-N-MC (Figure 5C) and A549 (Figure 5D) as indicated by fluorescence microscope and flow cytometry analysis. BBR treatment remarkably reduced the elevated ROS in the cells. In addition, BBR largely abolished the EV71-elevated cellular ROS level, reversed the decrease of antioxidant index SOD (Figure 5E) and increase of peroxidation index MDA (Figure 5F) in these cells. To confirm the key role of ROS production in EV71 replication, we treated the EV71-infected cells with 100 μM NAC, a commonly used antioxidant (ROS inhibitor). As shown in Figure 5G, NAC largely diminished the EV71 RNA content and VP1 protein expression in virus-infected cells. These results suggested that BBR inhibits EV71 replication by reducing cellular ROS production.

### Berberine inhibits EV71 replication by regulating Keap1-Nrf2 axis

**FIGURE 6:**
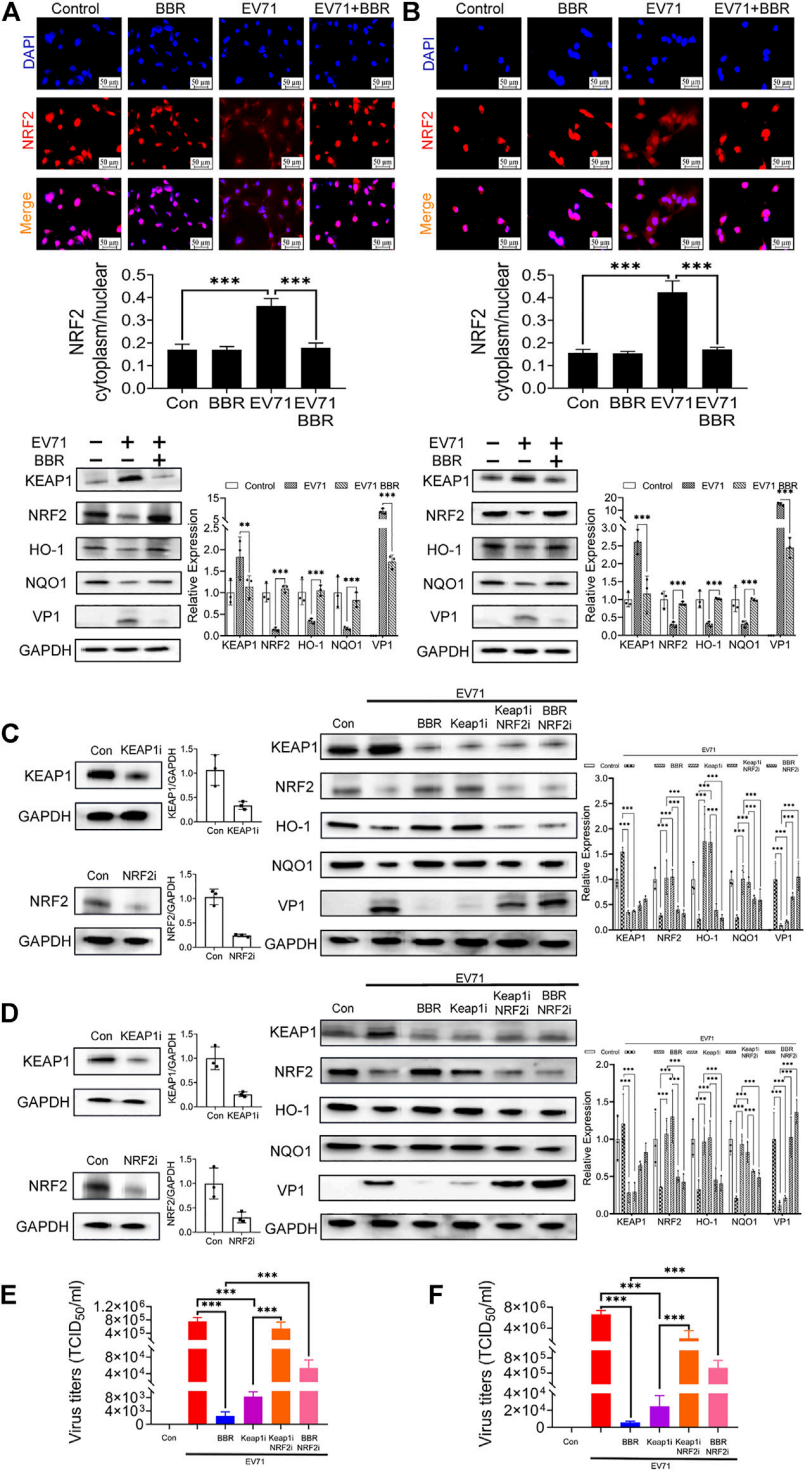
Effect of BBR in regulating Keap1-Nrf2 axis and downstream antioxidant proteins. Effects of BBR on the nuclear translocation of Nrf2 and expression of related proteins in **(A)** primary astrocytes and **(B)** U251 cells. Primary astrocytes and U251 were infected with EV71 (MOI = 1) for 2 h and then treated with BBR (5 μM) for 24 h. The cells were subjected to immunofluorescence staining with DAPI (Blue) and Nrf2 (Red) (*n* = 3). The red fluorescence intensity in cytoplasm and nucleus of these images was analyzed by High Content Image Processing and Analysis System, and the ratio was calculated. bar = 50 μm. The expression of Keap1, Nrf2, HO-1, NQO1, and EV71 VP1 proteins were examined by Western blot. The influence of Keap1 and/or Nrf2 knockdown on the antiviral efficacy of BBR in **(C,E)** primary astrocytes and **(D, F)** U251 cells. Western blot analysis was performed 24 h after siRNAs transfection in **(C)** primary astrocytes and **(D)** U251 cells. Virus titers were analyzed 72 h after transfection in **(E)** primary astrocytes and **(F)** U251 cells (*n* = 10). Data are presented as SD. Statistical significance was compared between two groups was represented by asterisks marked correspondingly in the figures, **p < 0.01, ***p < 0.001.

Nrf2 is a transcription factor that enhances the expression of antioxidant proteins such as HO-1 and NQO1 (Bai et al., 2020). Keap1 negatively regulates Nrf2 *via* ubiquitinization and degradation. To further explore the anti-EV71 mechanism of BBR, we analyzed the effects of BBR on the Keap1-Nrf2 axis and protein translocation of Nrf2 from cytoplasm to nucleus. As shown in Figure 6, EV71 infection resulted in remarkable repression of Nrf2 nuclear translocation. BBR restored the localization of Nrf2 in the nucleus and decreased the cytoplasm/nucleus ratio in primary astrocytes (Figure 6A) and U251 cell line (Figure 6B). Concurrently, BBR treatment reduced the protein expression of Keap1, increased the expression of Nrf2 and downstream antioxidant proteins HO-1 and NQO1, and inhibited EV71 replication in infected cells.

To verify the requirement of Keap1-Nrf2 axis in the anti-EV71 activity of BBR, we further analyzed the influence of Keap1 and/or Nrf2 knockdown on the antiviral efficacy of BBR in primary astrocytes (Figures 6C,E) and U251 cell line (Figures 6D,F). Both BBR treatment and Keap1 silencing increased the expression of Nrf2 and its downstream antioxidant proteins HO-1 and NQO1, and inhibited EV71 replication. However, such effects were abolished after Nrf2 is silenced. These data demonstrated that Keap1-Nrf2 axis plays a key role in the antioxidant and anti-EV71 activity of BBR.

## Discussion

EV71 is the etiologic pathogen for HFMD that is particularly associated with lethal neurological and systemic complications and threatens children’s life worldwide (Dai et al., 2017; Dai et al., 2019). Although significant efforts have been put on the discovery and development of antiviral agents toward EV71 infection, currently there is no approved antiviral therapies for EV71 infections (Kuo and Shih, 2013; Shang et al., 2013; Tan et al., 2014; Pourianfar and Grollo, 2015). In the present study, we demonstrated for the first time that BBR strongly inhibited EV71 replication in neural cells and prevented lethal neurological infection in newborn mice. There are several interesting findings on the antiviral effects and mechanisms of BBR against lethal EV71 infection.

In neural cell lines, dose-dependent analysis indicated much more potent antiviral activities of BBR against EV71 infection compared with antiviral drug Ribavirin (IC_50_ 2.79–4.03 μM vs. 53.82–58.18 μM). The anti-EV71 activities in neural cell lines are also significantly higher than in A549 cells (IC_50_ 6.83 μM) in our study and in Vero cells with IC_50_ ranging from 7.43 to 10.25 μM in different EV71 strains reported previously (Wang H. et al., 2017), suggesting that BBR has better antiviral effects on neural cells. Time of compound addition assay in different cell lines indicated that BBR targets the replication stage to prevent EV71 infection. These data revealed a preferential anti-EV71 activity of BBR in neural cells and implied more efficient underlying mechanisms against EV71 replication.

EV71-induced pulmonary edema in severe cases of HFMD has been considered neurogenic in origin, as it has been observed in encephalitis without signs of pneumonia and myocarditis (Huang et al., 2011). Neurogenic pulmonary edema (NPE) and subsequent rapid onset cardiopulmonary failure are hallmarks of EV71-induced mortality. It was speculated that systemic and local proinflammatory responses resulting from EV71-related inflammation and brain damage are involved in the development of pulmonary edema in EV71 patients (Du et al., 2019). Proinflammatory factors IL-6, TNF-α, IL-1β were found significantly higher in patients with encephalitis and NPE than those with mild disease (Jin et al., 2017; Jin et al., 2021b). In the EV71 lethal infection mouse model, we also found remarkably higher level of these proinflammatory factors in infected organs. BBR treatment significantly reduced the expression of the proinflammatory factors in brain and lungs, improved the clinical scores, alleviated EV71 pathological injuries of various major organs (including brain, lung, heart, liver and kidney), and improved survival of the infected newborn mice. These effects coincide with remarkably reduced EV71 virus replication in the infected organs, demonstrating a strong *in vivo* antiviral effect of BBR.

EV71 replication was detected largely in the brainstem of our newborn mouse model, which is consistent with lethal EV71 infection in patients (Xing et al., 2014). Immunofluorescence co-localization of EV71 VP1 protein with astrocyte marker GFAP, microglia marker IBA-1 and neuronal marker NeuN in brainstem cryosections indicated that EV71 mainly infected astrocytes, which is consistent with previous studies in human, rhesus macaque and mouse brain (Feng et al., 2016; Luo et al., 2019; Jin et al., 2021a). Meanwhile, we found that EV71 also infected most microglia in brainstem, albeit in fewer numbers than astrocytes. An interesting finding is that EV71-positive astrocytes are more distributed in the ventral zone of brainstem, where more severe pathological changes are seen (Figure 3B, Figure 4). The preferential EV71 infection of astrocytes and microglia may trigger strong inflammatory injury and impair host antiviral responses in brainstem. Primary culture of astrocytes and microglia (not shown) from newborn mouse brain confirmed the efficient EV71 replication that was mostly inhibited by BBR treatment. These data indicated that BBR efficiently suppressed EV71 replication mainly in glial cells in the brainstem of newborn mice and alleviated pathological injuries.

**FIGURE 7:**
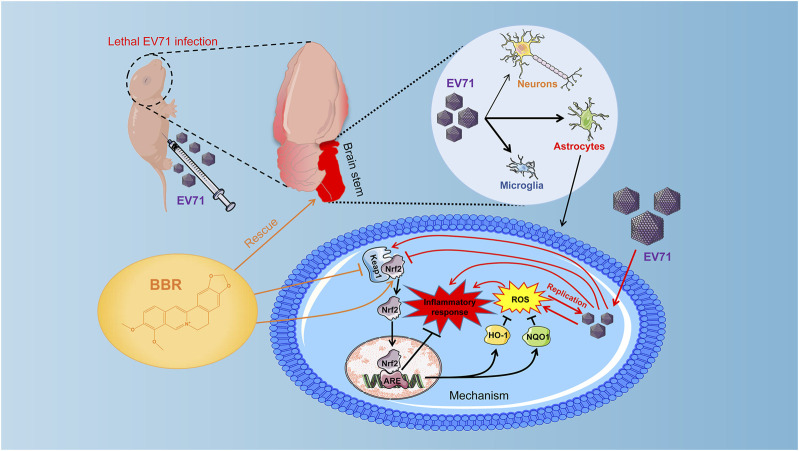
Effect and mechanisms of BBR against lethal EV71 neurological infection. EV71 infection was localized predominantly in the brainstem in the newborn mouse model. BBR prevents lethal EV71 neurological infection *via* inhibiting virus replication mainly in glial cells. BBR inhibits EV71-induced upregulation of Keap1, thereby enhances the expression and nuclear translocation of Nrf2, promotes expression of antioxidant enzymes HO-1 and NQO1, abolishes elevated ROS production, virus replication and inflammatory injury in the brain.

Previous studies suggested that the generation of reactive oxygen species (ROS) induced by EV71 infection is necessary for its replication in cells (Bai et al., 2020). EV71 can induce ROS formation through integrin beta1/EGFR-Rac1-dependent oxidative stress, attenuating ACOX1 production and peroxisome biogenesis, and reducing Nrf2 activation in different cell types (Tung et al., 2011; Cheng et al., 2014; Bai et al., 2020; You et al., 2020). The brain is inadequately equipped with antioxidant defense systems and prone to oxidative injury (Cobley et al., 2018), which facilitates EV71 replication. Indeed we found remarkably elevated cellular ROS levels that coincided with EV71 replication in primary cultured astrocytes and various cell lines. BBR efficiently abolished the virus-elevated ROS production and greatly inhibited EV71 replication by up-regulating Nrf2 *via* the Keap1-Nrf2 axis in these cells. The nuclear localization of Nrf2 and expression of downstream antioxidant enzymes HO-1 and NQO1 were also increased by BBR treatment, further supporting the antioxidant mechanism (Figure 7). The inhibitory effects of BBR on virus replication in neural cells (IC_50_ < 4.03 μM) are apparently more potent than in A549 cells (IC_50_ 6.83 μM) in our study and in Vero cells (IC_50_ ranging from 7.43 to 10.25 μM in different EV71 strains) reported previously (Wang H. et al., 2017). Other antiviral mechanisms of BBR may be involved such as suppressing the EV71-induced autophagy by activating AKT and inhibiting the phosphorylation of JNK and PI3K (Wang H. et al., 2017). This suggests that BBR can inhibit EV71 infection through mechanisms other than the Keap1-Nrf2 axis. Based on the effect of Nrf2 knockdown on EV71 virus titers after BBR treatment (about 100 times in U251 and more than 20 times in primary astrocytes), the Nrf2 dependent pathway seems more significant for BBR anti-EV71 activity (Figures 6E,F). In addition, the western blot results also indicate that in the early stage (24 h) of EV71 infection, BBR relies more on the Keap1-Nrf2 axis to inhibit EV71 replication (Figures 6C,D). Our results revealed that modulating antioxidant responses may be a more efficient mechanism for BBR to prevent neuropathic EV71 infection.

## Conclusion

In conclusion, the present study demonstrated that BBR prevents lethal EV71 neurological infection *via* inhibiting virus replication through regulating Keap1-Nrf2 axis and reducing elevated ROS generation in infected neural cells. The study may provide a potential antiviral treatment for severe EV71 infection associated with neurological complications.

## Data Availability

The original contributions presented in the study are included in the article/Supplementary Material; further inquiries can be directed to the corresponding author.

## Abbreviations

EV71, Enterovirus 71; HFMD, Hand, foot, and mouth disease; BBR, Berberine; Nrf2, Nuclear factor (erythroid-derived 2)-like 2; Keap1, Kelch-like ECH-associated protein 1; ROS, Reactive oxygen species; AM, Aseptic meningitis; BSE, Brain stem encephalitis; AFP, Acute flaccid paralysis; NPE, Neurogenic pulmonary edema; CPE, Cytopathic effect; OS, Oxidative stress; PE, Pulmonary edema; BBB, Blood-brain barrier; DEs, Detoxifying enzymes; AEs, Antioxidant enzymes.
