# Supplementary material for: Berberine prevents lethal EV71 neurological infection in newborn mice
Source: Front Pharmacol. 2022 Oct 25;13:1027566. doi: 10.3389/fphar.2022.1027566 (PMC9640474; doi:10.3389/fphar.2022.1027566)
Supplement: Supplementary file 1 [file DataSheet1.doc]

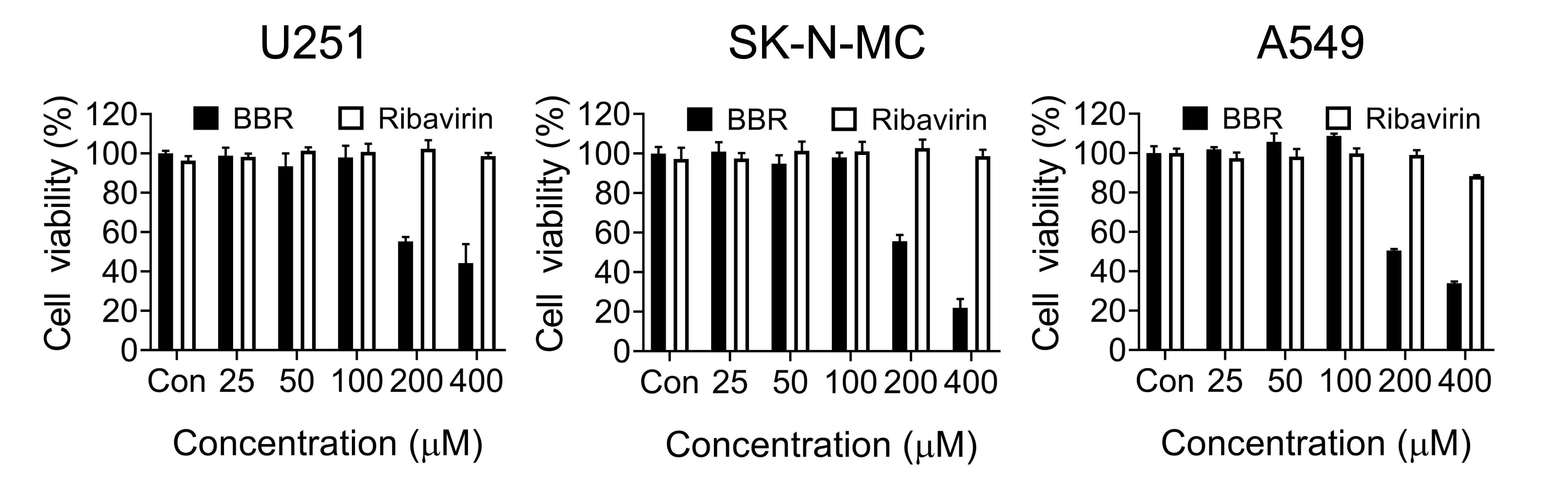


**FIGURE S1** The cytotoxicity of BBR in different cell lines. U251, SK-N-MC and A549 cells were incubated with BBR or ribavirin at different concentrations for 72 h. The cytotoxicity was measured by CCK-8 assay (n=6).

**TABLE S1** Therapeutic index (SI) of BBR and ribavirin for EV71 infection.

| **Cells** | **Compd.** | **CC_50_ (μM)** | **IC_50_ (μM)** | **SI** |
| --- | --- | --- | --- | --- |
| **U251** | BBR | 305.4 | 2.79 | 109.38 |
|  | Ribavirin | 940 | 58.18 | 16.16 |
| **SK-N-MC** | BBR | 232.4 | 4.03 | 57.72 |
|  | Ribavirin | 927.5 | 53.82 | 17.23 |
| **A549** | BBR | 255.9 | 6.83 | 37.47 |
|  | Ribavirin | 1491 | 30.30 | 49.21 |

CC_50_ of BBR or ribavirin were calculated by linear regression analysis of the cytotoxicity curves, and IC_50_ of BBR or ribavirin were calculated by linear regression analysis of the viral inhibition curves. SI was defined as the ratio of CC_50_ to IC_50_ (CC_50_/IC_50_).

TABLE S2 List of primers used for qPCR.

| Primer title | Orientation |
| --- | --- |
| qmIL-1β F | 5’-TCGCTCAGGGTCACAAGAAA-3’ |
| qmIL-1β R | 5’-CATCAGAGGCAAGGAGGAAAAC-3’ |
| qmIL-6 F | 5’-CAGGCGGTGCCTATGTCTC-3’ |
| qmIL-6 R | 5’-CGATCACCCCGAAGTTCAGTAG-3’ |
| qmTNFα F | 5’-ACGTGGAACTGGCAGAAGAG-3’ |
| qmTNFα R | 5’-CTCCTCCACTTGGTGGTTTG-3’ |
| qmGAPDH F | 5’-TGACCTCAACTACATGGTCTACA-3’ |
| qmGAPDH R | 5’-CTTCCCATTCTCGGCCTTG-3’ |
| EV71-5'UTR F | 5’-TCCTCCGGCCCCTGA-3’ |
| EV71-5'UTR R | 5’-AATTGTCACCATAAGCAGCCA-3’ |
| qhIL-1β F | 5’-ATGATGGCTTATTACAGTGGCAA-3’ |
| qhIL-1β R | 5’-GTCGGAGATTCGTAGCTGGA-3’ |
| qhIL-6 F | 5’-ACTCACCTCTTCAGAACGAATTG-3’ |
| qhIL-6 R | 5’-CCATCTTTGGAAGGTTCAGGTTG-3’ |
| qhTNFα F | 5’-CCTCTCTCTAATCAGCCCTCTG-3’ |
| qhTNFα R | 5’-GAGGACCTGGGAGTAGATGAG-3’ |
| qhGAPDH F | 5’-GGAGCGAGATCCCTCCAAAAT-3’ |
| qhGAPDH R | 5’-GGCTGTTGTCATACTTCTCATGG -3’ |

The sequences of primers involved in this study are listed. F, forward; R, reverse; qh (qm), the primers for human (mouse) genes used in quantitative real-time PCR.
